# Confidence, attitude, and practice of scientific research among health professions’ students in the United Arab Emirates

**DOI:** 10.1371/journal.pone.0304357

**Published:** 2024-05-31

**Authors:** Anan S. Jarab, Walid Al-Qerem, Karem H. Alzoubi, Shrouq R. Abu Heshmeh, Mays Hayek, Yazid N. Al Hamarneh, Salahdein Aburuz

**Affiliations:** 1 College of Pharmacy, Al Ain University, Abu Dhabi, United Arab Emirates; 2 AAU Health and Biomedical Research Center, Al Ain University, Abu Dhabi, United Arab Emirates; 3 Department of Clinical Pharmacy, Faculty of Pharmacy, Jordan University of Science and Technology, Irbid, Jordan; 4 Department of Pharmacy, Faculty of Pharmacy, Al-Zaytoonah University of Jordan, Amman, Jordan; 5 Department of Pharmacy Practice and Pharmacotherapeutics, University of Sharjah, Sharjah, United Arab Emirates; 6 Faculty of Pharmacy, Jordan University of Science and Technology, Irbid, Jordan; 7 Department of Pharmacology, Faculty of Medicine and Dentistry, University of Alberta, Edmonton, Alberta, Canada; 8 Department of Pharmacology and Therapeutics, College of Medicine and Health Sciences, The United Arab Emirates University, Al Ain, United Arab Emirates; International Medical University, MALAYSIA

## Abstract

**Objective:**

This study aimed to assess the confidence, attitude, and scientific research practices of undergraduate students of different health profession specialties.

**Methods:**

In this cross-sectional study, an online-based questionnaire was distributed as a Google Form via groups and pages of medical universities available on social media sites such as Facebook, WhatsApp, and Twitter to the second- to sixth-year students of different health profession specialties in different universities across the United Arab Emirates (UAE) in the period from October through December 2023 using the convenience sampling technique. The questionnaire included four parts that assessed socio-demographics and custom-designed research-related questions (6 items), perceived confidence (8 items), attitudes (14 items), and the practice in the context of scientific research and its implementation (9 items). Multivariate logistic regression analyses were conducted to explore the variables associated with the study outcomes, including confidence, attitudes, and practice levels.

**Results:**

The study included 522 undergraduate students. The participants reported low confidence, a negative attitude, and low scientific research practice. Regression results revealed that individuals without prior research experiences were less likely to have high confidence and practice compared to those with previous research experience (OR = 0.634, 95% CI: 0.426–0.945, p = 0.025; and OR = 0.139, 95%Cl: 0.090–0.216, P<0.001, respectively). Additionally, participants who reported difficulty in differentiating between various literature resources were less likely to have high confidence and practice compared to those who reported the ability to differentiate (OR = 0.627, 95% CI: 0.42–0.935, p = 0.022, and OR = 0.370, 95%Cl: 0.237–0.579, p<0.001, respectively). Furthermore, individuals who had not taught research methods in their undergraduate studies were less likely to have high practice (OR = 0.505, 95%Cl: 0.309–0.823, p = 0.06).

**Conclusions:**

Undergraduates of different medical specialties in the UAE demonstrated acceptable levels of confidence and attitude toward scientific research, with several areas for practice improvement. Education and training courses focusing on various aspects of scientific research should be incorporated into the medical curricula in order to enhance students’ confidence and practice of scientific research.

## Introduction

Research is an investigative process that aims to solve issues, increase knowledge, and improve our comprehension of the world. In order to create new concepts, technologies, and innovations as well as advance society, research is crucial [1]. In the healthcare industry, discovering treatments and cures for illnesses is a crucial aspect of medical research and could enhance patients’ quality of life. Research also helps medical professionals stay current on the newest developments and methods in their fields in order to provide the best care possible for patients [1].

Over the last few decades, there has been a discernible shift in undergraduate education to incorporate research aspects in medical and pharmacy schools [2–5].

In the United Arab Emirates (UAE), the goal of the colleges of medicine and other health sciences is to promote excellence in healthcare, medical education, and research. Such colleges are recognized as icons of knowledge and creativity in the medical field and are committed to generating highly qualified and caring healthcare professionals. Emphasizing research and the cultivation of new researchers are key priorities in these colleges’ educational missions [6]. In addition, undergraduate students in various healthcare specializations at UAE universities must complete research projects as a requirement for graduation from the respective colleges of specialization.

Students’ interest in conducting research, presenting their findings, and publishing their work at the national and international levels could be sparked by these developments. A student’s capacity for conducting scholarly research adds value to their academic progress by fostering the development of critical thinking and analytical abilities, as well as understanding and analyzing the tenets of evidence-based medicine [7–9]. Numerous studies have demonstrated the strong correlation between undergraduate research experience and future professional success as well as scholarly research activities [9–11].

Students majoring in health professions such as medicine comprise a group that could be future leaders in clinical research [12]. Therefore, investigating how medical students feel about scientific research is important. In addition to attitude, practice sharpens the skills necessary for success, and confidence gives one the courage to embark on research projects. Together, they create a cohesive group that not only equips students to meet the demands of modern healthcare but also positions them as agents of change and advancement in the medical field. This combination served as the inspiration for conducting this study.

The aim of the current study was (1) to assess health profession students’ confidence, attitude, and practice regarding scientific research and (2) to explore the factors associated with evaluated confidence attitudes and practices.

## Methods

An online-based questionnaire was distributed as a Google Form via groups and pages of medical universities available on social media sites such as Facebook, WhatsApp, and Twitter.

### Study design and subjects

In this cross-sectional study (S1 Checklist. Strobe Checklist), a validated questionnaire was distributed as a Google Form via groups and pages of medical universities available on social media sites such as Facebook, WhatsApp, and Twitter to students in different colleges that offer health programs in the UAE, including medicine, dentistry, pharmacy, doctor of pharmacy, nursing, and applied medical sciences, using convenience sampling in the period from October through December 2023. The indicated groups and social media pages included only medical specialty students from the indicated universities or colleges, as membership in these groups is restricted to students from that particular university and medical specialty. Additionally, a question ensuring the field of the study, listing only students in the health profession fields, were included in the survey to ensure the eligibility of the respondents. The included universities were United Arab Emirates University, Al Ain University, the University of Sharjah, Ajman University, Mohammed Bin Rashid University of Medicine and Health Sciences, Rak Medical and Health Sciences University, and Gulf Medical University. The exclusion criteria were first-year students who studied outside the health school campus, as well as postgraduate students.

### Study instrument

A comprehensive review of the literature was conducted before developing an online, self-administered survey (S1 File. Study Survey) for the current study [2,13–18]. The sociodemographic data were collected in the first section of the questionnaire. The second 8-item part of the questionnaire assessed students’ confidence in utilizing and implementing scientific research on a 4-point Likert scale ranging from “not at all” (1 point) to “extensively” (4 points). Then, the scores were summed up to calculate the confidence score for each participant, with a maximum possible score of 32. In the third part, 14 questions were used to assess students’ attitudes toward scientific research on a 5-point Likert scale ranging from “strongly disagree” (1 point) to “strongly agree” (5 points). Then, the scores were summed up to calculate the attitude score for each participant, with a maximum possible score of 70. The final part of the questionnaire assessed the practice of scientific research using nine questions with “yes” or “no” responses, with each “yes” response corresponding to 1 point. A group of experts, including academic professors of different health specialties, evaluated the questionnaire for content validity to ensure its relevancy and comprehensiveness. Regarding face validity, the questionnaire was piloted with ten students of different health specialties to ensure its appropriateness, clarity, and feasibility. None of the data from the pilot test was incorporated into the final data analysis. Cronbach’s alpha values for the confidence, attitude, and practice domains were 0.88, 0.83, and 0.86, respectively.

### Ethical approval

The current research received the required ethical approval from the research ethics committee at Al Ain University- Abu-Dhabi Campus (Ref. No. COP/AREC/AD/14). Before filling out the study questionnaire, the participants were asked to tick a box to indicate informed consent to participate in the study.”

### Sample size calculation

When applying the Krejcie & Morgan equation to calculate the required minimum sample size [19], the equation revealed that for an indefinite population with a 95% significance level, a 5% margin of error, and a 50% population proportion, the minimal required sample was 385.

### Statistical analysis

All statistical analyses were conducted using the Statistical Package for the Social Sciences (SPSS, version 28). Categorical variables were presented as frequencies and percentages, and continuous variables were presented as medians and 95% Cl. The Q-Q plots indicated the data was not normally distributed; therefore, nonparametric analyses were conducted. Cronbach’s alphas were computed to evaluate the internal consistency of the questionnaire’s three domains. A bivariate analysis was conducted to explore the association between different sociodemographic variables and each study outcome, including confidence, attitude, and practice. For the purpose of regression analysis, the outcomes’ scores were dichotomized as high or low based on the median score of each outcome. A chi-square test was performed to assess the association between each scale level and different demographic characteristics. Multivariate logistic regression analyses were conducted to explore the variables associated with the study outcomes, including confidence, attitudes, and practice levels. The significant level was determined as <0.05.

## Results

The study included 522 participants. The median age of the participants was 22 (22–23) years. The majority of the participants were females (76.1%), studied in private universities (76.8%), had been taught research methods during their undergraduate studies (66.9%), could differentiate between different literature resources (61.5%), and had no prior research experience (50.6%) (Table 1).

**Table 1:** Sociodemographic characteristics of the enrolled participants.

		Frequency (%)
Age		22 (22–23)
Gender	Female	397 (76.1%)
	Male	125 (23.9%)
Type of university	Private	401 (76.8%)
	public	121 (23.2%)
Have you been taught “research methods” during your undergraduate studies?	No	173 (33.1%)
	Yes	349 (66.9%)
Do you have prior research experience, or have you been involved in conducting research before?	No	264 (50.6%)
	Yes	258 (49.4%)
Can you differentiate between different literature resources?	No	201 (38.5%)
	Yes	321 (61.5%)

Table 2 demonstrates participants’ responses to the confidence items. The results indicated that the tasks with the highest confidence level were “discussing the research study findings” (33.9%), “interpreting study findings” (31%), and “implementing experiments and collecting data’ (30.3%). On the other hand, the task with the lowest confidence level was “making a critical appraisal of the literature,” followed by “searching literature for information” (18.6% and 19.2% respectively). The median for the confidence score was 24 (24–25) out of a maximum possible score of 32. The median for all confidence items was 3, and the lowest (25–75) percentile was (2–3) for the items “searching the literature for information” and “making a critical appraisal of the literature”.

**Table 2:** Participants’ responses to the confidence items.

	Not at all	Limited	Somewhat	Extensively	
	Frequency (%)	Frequency (%)	Frequency (%)	Frequency (%)	Median (25–75)
Searching the literature for information	41 (7.9%)	160 (30.7%)	221 (42.3%)	100 (19.2%)	3 (2–3)
Creating a research question or specifying study objective	24 (4.6%)	124 (23.8%)	231 (44.3%)	143 (27.4%)	3 (2–4)
Selecting the appropriate research instruments and methods	29 (5.6%)	126 (24.1%)	221 (42.3%)	146 (28%)	3 (2–4)
Implementing experiments and collecting data	29 (5.6%)	134 (25.7%)	201 (38.5%)	158 (30.3%)	3 (2–4)
Conducting data analysis.	42 (8%)	152 (29.1%)	195 (37.4%)	133 (25.5%)	3 (2–4)
Interpreting study findings	30 (5.7%)	110 (21.1%)	220 (42.1%)	162 (31%)	3 (2–4)
Discussing the research study findings	29 (5.6%)	110 (21.1%)	206 (39.5%)	177 (33.9%)	3 (2–4)
Making a Critical appraisal of the literature	65 (12.5%)	153 (29.3%)	207 (39.7%)	97 (18.6%)	3 (2–3)

Table 3 presents the participants’ responses to the attitude items. Among the statements that were reverse coded, the statement with the highest percentage of agreement was " undertaking research increases the burden on already overworked students/trainees" (41.2%), followed by the statement " conduction of research is difficult" (32.8%), and the statements with the lowest percentage of agreement were “negative effects of scientific research exceed positive ones.” (9.9%) and “I find it difficult to understand the concepts of research.” (15.9%). Regarding the remaining statements, the statement with the highest percentage of participants who disagreed or strongly disagreed was "I am inclined to learn about scientific research principles" (15.2%). while the statements with the lowest percentage of disagree/strongly disagree responses were "patient outcome improves with continued medical research" (2.5%), "use of research-based evidence is the basis of medical progress" (2.7%), and "valid discoveries are impossible without scientifically sound research" (2.9%). The median for the attitude items was 56 (55–57) out of a maximum possible score of 70. The attitude median ranged between 2 and 5, with the lowest median reported being 2 (1–3) for “I don’t feel confident to participate in a scientific research project.”

**Table 3:** Participants’ responses to the attitude items.

	Strongly agree	Agree	Neutral	Disagree	Strongly disagree	
	Frequency (%)	Frequency (%)	Frequency (%)	Frequency (%)	Frequency (%)	Median (25–75)
I find it difficult to understand the concepts of research. *	29 (5.6%)	54 (10.3%)	226 (43.3%)	159 (30.5%)	54 (10.3%)	3 (2–3)
Conduction of research is difficult *	48 (9.2%)	123 (23.6%)	211 (40.4%)	102 (19.5%)	38 (7.3%)	3 (2–4)
I don’t feel confident to participate in a scientific research project. *	43 (8.2%)	66 (12.6%)	125 (23.9%)	139 (26.6%)	149 (28.5%)	2 (1–3)
Undertaking research increases the burden on already overworked students/trainees. *	87 (16.7%)	128 (24.5%)	141 (27%)	114 (21.8%)	52 (10%)	4 (3–5)
Negative effects of scientific research exceed positive ones. *	21 (4%)	31 (5.9%)	114 (21.8%)	130 (24.9%)	226 (43.3%)	3 (2–4)
I am inclined to learn about scientific research principles.	182 (34.9%)	131 (25.1%)	130 (24.9%)	63 (12.1%)	16 (3.1%)	4 (3–5)
Awareness of scientific research principles is essential for obtaining accurate and objective data.	298 (57.1%)	118 (22.6%)	78 (14.9%)	21 (4%)	7 (1.3%)	5 (4–5)
Scientific research facilitates a better understanding of problems.	254 (48.7%)	157 (30.1%)	80 (15.3%)	26 (5%)	5 (1%)	4 (4–5)
Research is useful for my future career.	296 (56.7%)	123 (23.6%)	71 (13.6%)	22 (4.2%)	10 (1.9%)	5 (4–5)
Medical students should learn and participate in scientific research during university education.	290 (55.6%)	129 (24.7%)	83 (15.9%)	13 (2.5%)	7 (1.3%)	5 (4–5)
Every healthcare provider must be aware of scientific research principles.	257 (49.2%)	150 (28.7%)	88 (16.9%)	21 (4%)	6 (1.1%)	4 (4–5)
Patient outcome improves with continued medical research.	297 (56.9%)	146 (28%)	66 (12.6%)	10 (1.9%)	3 (0.6%)	5 (4–5)
Use of research-based evidence is the basis of medical progress.	283 (54.2%)	139 (26.6%)	86 (16.5%)	12 (2.3%)	2 (0.4%)	5 (4–5)
Valid discoveries are impossible without scientifically sound research.	243 (46.6%)	157 (30.1%)	107 (20.5%)	10 (1.9%)	5 (1%)	4 (4–5)

* Reverse coding.

As shown in Table 4, the practice that was most commonly reported among participants was conceptualizing a research idea (52.1%), followed by “implementing research experiment or collecting research data” (51.1%), while the least reported practices were “preparing a research manuscript for submission in a scientific journal” (20.9%) and “writing a research manuscript” (28.4%). The median for the practice score was 3 (3–4) out of a maximum possible score of 9.

**Table 4:** Participants’ responses to the practice items.

	No	Yes
	Frequency (%)	Frequency (%)
Participation in scientific research workshop	317 (60.7%)	205 (39.3%)
Conducting literature review	347 (66.5%)	175 (33.5%)
Conceptualizing a research idea	250 (47.9%)	272 (52.1%)
Writing a research proposal	280 (53.6%)	242 (46.4%)
Implementing research experiment or collecting research data	255 (48.9%)	267 (51.1%)
Analyzing data	280 (53.6%)	242 (46.4%)
Preparing an abstract for presentation in a conference	335 (64.2%)	187 (35.8%)
Writing a research manuscript	374 (71.6%)	148 (28.4%)
Preparing a research manuscript for submission in a scientific journal	413 (79.1%)	109 (20.9%)

The bivariate analysis showed that the participants who had been taught research methods during their undergraduate study (median = 24 vs.22, p = 0.021), those who had prior research experience or were involved in conducting research before (median = 24 vs.23, p<0.01) and those who were able to differentiate between different literature resources (median = 24 vs.22, p = <0.01) had significantly higher confidence scores when compared with their counterparts (Table S1 in S2 File). The participants who attended a private university had a higher practice score compared with those who attended a public university (median = 3 vs.2, p = 0.037). Moreover, the participants who had been taught research methods during their undergraduate study (median = 4 vs.1, p<0.01), those who had prior research experience or had been involved in conducting research before (median = 5 vs.1, p<0.01) and those who were able to differentiate between different literature resources (median = 4 vs.1, p<0.01) had significantly higher practice scores than their counterparts (Table S2 in S2 File). There were no significant differences between the evaluated sociodemographic characteristics and the attitude level (Table S3 in S2 File).

The multivariate regression analysis (Table 5) showed no significant association between the independent variables and attitude level. In terms of confidence, students without prior research experiences (OR = 0.634, 95% CI: 0.426–0.945, P<0.05), and those who reported difficulty differentiating between various literature resources (OR = 0.627; 95% CI: 0.42–0.935; P<0.05) were less likely to have high confidence to implement scientific research. Students who had not been taught research methods (OR = 0.505; 95%Cl: 0.309–0.823; P<0.05), had no prior research experiences (OR = 0.139; 95%Cl: 0.090–0.216; P<0.001) and those who reported difficulties in differentiating between literature resources (OR = 0.370, 95%Cl: 0.237–0.579; P<0.01) had lower odds of having a high practice level.

**Table 5:** Multivariate logistic regression of different sociodemographic variables with confidence, practice and attitude levels.

	Practice score				Confidence score				Attitude score			
	p-value	OR	95% Confidence Interval for OR		p-value	OR	95% Confidence Interval for OR		p-value	OR	95% Confidence Interval for OR	
			Lower Bound	Upper Bound			Lower Bound	Upper Bound			Lower Bound	Upper Bound
Intercept	<0.001				<0.001				<0.001			
Age	0.796	1.011	0.929	1.100	0.898	0.995	0.925	1.071	0.796	1.011	0.929	1.100
Gender	0.123	0.676	0.411	1.112	0.478	1.169	0.759	1.799	0.123	0.676	0.411	1.112
Type of university	0.984	1.005	0.597	1.693	0.522	1.157	0.741	1.806	0.984	1.005	0.597	1.693
Have you been taught research methods during your undergrad study?	0.006	0.505	0.309	0.823	0.518	0.867	0.562	1.337	0.006	0.505	0.309	0.823
Do you have prior research experience, or have you been involved in conducting research before?	<0.001	0.139	0.090	0.216	0.025	0.634	0.426	0.945	<0.001	0.139	0.090	0.216
Can you differentiate between different literature resources?	<0.001	0.370	0.237	0.579	0.022	0.627	0.421	0.935	<0.001	0.370	0.237	0.579
Constant score	0.176	4.002			0.828	0.825			0.176	4.002		

## Discussion

The current study showed a potential for improvement in confidence, attitude, and practice of scientific research among health profession students in Emirati universities. Lack of prior research experience and difficulties in differentiating literature resources were associated with lower confidence and practice in scientific research. Additionally, the absence of education on research methods during undergraduate studies was linked to reduced research practice.

The study demonstrated a window for confidence improvement in various aspects of research implementation, starting with discussing and interpreting research findings, selecting appropriate research methods, creating a research question or specifying the study objective, conducting data analysis, and conducting a literature review to critically evaluate the existing literature. Nonetheless, the least amount of confidence was expressed in finding information through a literature search (19.2%) and critically evaluating the literature (18.6%). These findings highlight the pressing need to enhance health profession students’ confidence in the core elements of scientific research, particularly in the realms of literature search and critical literature evaluation. Therefore, academic institutions, funding agencies, and research communities in the UAE should consider the implementation of tailored training and educational programs through workshops, seminars, or online courses focusing on key areas of research [20].

The participants in the current study demonstrated a higher attitude towards scientific research than medical students who participated in an Egyptian study [21]. On the other hand, a study conducted in the Gulf states of Saudi Arabia, Kuwait, and Bahrain found that medical students exhibited a low level of knowledge but maintained an overall positive attitude toward scientific research [2]. Other studies have also reported better attitude levels towards scientific research among medical [22–26] and dental students [22]. Given that more than a third of participants in the current study believed that conducting research increases the burden on already overburdened students or trainees (41.2%), and that conducting research is perceived as difficult (32.8%), it is obvious that the academic curricula in the UAE’s medical schools need to be modified in a balanced manner that allows for the inclusion of research courses without further escalating the burden on students. According to an Indian study, the majority of participating medical students strongly supported the inclusion of research in the undergraduate curriculum and thought that it would aid in improving understanding and practice [24]. Educational institutions can create a smoother and more encouraging environment for students and make research accessible and an essential part of their academic journey by facilitating the incorporation of research topics into the curriculum. On the other hand, a mere 15.2% indicated no interest in acquiring knowledge about the fundamentals of scientific research, making it imperative to concentrate on understanding and addressing the reasons behind this lack of interest.

In the current study, students demonstrated insufficient practice in the majority of research-related areas, with writing research manuscripts (28.4%) and getting them ready to be submitted to scientific journals (20.9%) demonstrating the least practiced area. Similar results were reported in a previous study conducted among medical students in Yemen [23]. While 97.1% of participants in another study of senior medical students in Saudi Arabia agreed that research is important in the medical field, only half of them conducted research as part of their academic studies [10]. Another Saudi study found that, despite having a positive attitude toward research, only 38.1% of the medical students and interns participated in it [27]. The observed disappointing rate of research practice among these students sheds light on the importance of enhancing research learning and bridging the gap between research education and practical application among medical students in the UAE. This can be achieved by including interactive classroom discussions, which will give students an environment for group exploration and the application of various research ideas [28]. Research training courses are also essential for improving students’ skills in different areas of research, particularly research paper writing, and preparing them for publication in scientific journals.

The current study revealed that individuals without prior research experience exhibited lower confidence levels and less practice in scientific research. This finding is justifiable, as exposure to various aspects of research enhances students’ confidence and improves their proficiency in conducting scientific research [29]. Furthermore, participants struggling to differentiate between literature resources reported lower confidence and less practice in scientific research, likely stemming from their limited experience in this area. Moreover, the study found significantly lower practice levels in students who had not been taught research methods during their undergraduate studies. This result can be attributed to the lack of education and training in research methods, affecting their application and leading to reduced practice. These findings shed light on the importance of incorporating educational courses regarding various research aspects during undergraduate studies in medical colleges across the UAE and developing tailored training programs to enhance students’ expertise, thereby improving their confidence and proficiency in scientific research.

The current study has some limitations. The convenience sampling used in this study might cause selection bias and affect the generalizability of the findings. The cross-sectional study design cannot confirm the cause-effect relationship. Using a self-report survey may expose the responses to social-desirability bias, affecting their accuracy. As the questionnaire was distributed online, we cannot ensure that the same respondent did not complete it more than once. Although the questionnaire was distributed via members-restricted groups and pages of medical universities on social media at different colleges, where membership in these groups was allowed only for students from that particular university and medical specialty, we cannot entirely exclude the remote possibility that members of these groups could have forwarded the questionnaire link to others outside the groups who are not students with one of the medical specialties. However, to avoid such unintentional participation and ensure the eligibility of all the respondents, we included a question emphasizing that that only students within the health profession fields are enrolled.

## Conclusions

Undergraduate students of different health specialties demonstrated a margin for improvement in confidence, attitude, and scientific research practices in the UAE. Research confidence and practice were found to be lower in those without previous research experience and with difficulty differentiating literature resources. Furthermore, there was a correlation between undergraduate education lacking in research methodology and reduced research practice. Future educational programs should include education and training courses concerning various areas of research in medical colleges’ undergraduate curricula to boost students’ confidence and practice carrying out various research assignments.

## Supporting information

S1 Checklist STROBE statement—Checklist of items that should be included in reports of *cross-sectional studies*. (DOCX)

S1 File Study survey. (DOCX)

S2 File (DOCX)
