# Supplementary material for: Confidence, attitude, and practice of scientific research among health professions’ students in the United Arab Emirates
Source: PLoS One. 2024 May 31;19(5):e0304357. doi: 10.1371/journal.pone.0304357 (PMC11142569; doi:10.1371/journal.pone.0304357)
Supplement: S1 File — (DOCX) [file pone.0304357.s002.docx]

Dear participant,

If you are a college undergraduate student living in the United Arab Emirates and studying a health/medical specialty, you are kindly invited to participate in this study conducted by a group of academic researchers to assess the confidence, attitude, and practice toward scientific research among students in medical colleges. All the information gathered is anonymous and will be treated confidentially. Your participation in this study is voluntary. Completing the questionnaire requires only 10 to 15 minutes. Thank you in advance for your time and participation.

Please do not complete the survey if you are a first-year student or postgraduate (Master/PhD) student

Thank you

**I have read the study instructions and I agree to participate in the study □**

Demographics:

**Age ---------------**

**Gender**

- Male
- Female

**Type of university**

- Government/ public
- Private

**University**

- United Arab Emirates University
- Al Ain University
- Mohammed Bin Rashid University of Medicine and Health Sciences
- Gulf Medical University
- Ajman University
- Rak Medical and Health Sciences University
- University Of Sharjah

**College**

- Medicine
- Dentistry
- Pharmacy
- Doctor of Pharmacy
- Nursing
- Applied medical sciences

**Academic year**

- 2nd year
- 3rd year
- 4th year
- 5th year
- 6th year

**Have you been taught research methods during your undergraduate study?**

- Yes
- No

**Do you have a prior research experience, or have you been involved in conducting research before?**

- Yes
- No

**Can you differentiate between different literature resources?**

- Yes
- No

**Confidence**

Please indicate how much confident you are to perform each of the following research-related tasks:

**1. Searching literature for information**

- Not at all
- Limited
- Somewhat
- Extensively

**2. Creating a research question or specifying study objective**

- Not at all
- Limited
- Somewhat
- Extensively

**3. Selecting the appropriate research instruments and methods.**

- Not at all
- Limited
- Somewhat
- Extensively

**4. Implementing experiments and collecting data**

- Not at all
- Limited
- Somewhat
- Extensively

**5. Conducting data analysis**

- Not at all
- Limited
- Somewhat
- Extensively

**6. Interpreting study findings**

- Not at all
- Limited
- Somewhat
- Extensively

**7. Discussing the research study findings.**

- Not at all
- Limited
- Somewhat
- Extensively

**8. Making Critical appraisal of the literature**

- Not at all
- Limited
- Somewhat
- Extensively

**Attitude**

**1. I find it difficult to understand the concepts of research.**

- Strongly disagree
- Disagree
- Neutral
- Agree
- Strongly agree

**2. Conduction of research is difficult**

- Strongly disagree
- Disagree
- Neutral
- Agree
- Strongly agree

**3. I do not feel confident to participate in a scientific research project.**

- Strongly disagree
- Disagree
- Neutral
- Agree
- Strongly agree

**4. Undertaking research increases burden on already overworked students/trainees.**

- Strongly disagree
- Disagree
- Neutral
- Agree
- Strongly agree

**5. Negative effects of scientific research exceed positive ones.**

- Strongly disagree
- Disagree
- Neutral
- Agree
- Strongly agree

**6. 4. I am inclined to learn about scientific research principle.**

- Strongly disagree
- Disagree
- Neutral
- Agree
- Strongly agree

**7. Awareness of scientific research principles is essential for obtaining accurate and objective data.**

- Strongly disagree
- Disagree
- Neutral
- Agree
- Strongly agree

**8. Scientific research facilitates better understanding of problems.**

- Strongly disagree
- Disagree
- Neutral
- Agree
- Strongly agree

**9. Research is useful for my future career**

- Strongly disagree
- Disagree
- Neutral
- Agree
- Strongly agree

**10. Medical students should learn and participate in scientific research during university education.**

- Strongly disagree
- Disagree
- Neutral
- Agree
- Strongly agree

**11. Every healthcare provider must be well acquainted with the scientific research principles.**

- Strongly disagree
- Disagree
- Neutral
- Agree
- Strongly agree

**12. Patient outcome improves with continued medical research**

- Strongly disagree
- Disagree
- Neutral
- Agree
- Strongly agree

**13. Use of research-based evidence is the basis of medical progress.**

- Strongly disagree
- Disagree
- Neutral
- Agree
- Strongly agree

**14. Valid discoveries are impossible without scientifically sound research.**

- Strongly disagree
- Disagree
- Neutral
- Agree
- Strongly agree

**Practice**

**Have you ever participated in:**

**1. Scientific research workshop?**

- Yes
- No

**2. Conducting literature review**

- Yes
- No

**3. Conceptualizing a research idea**

- Yes
- No

**4. Writing a research proposal**

- Yes
- No

**5. Implementing research experiment or collecting research data?**

- Yes
- No

**6. Analyzing data**

- Yes
- No

**7. Preparing an abstract for presentation in a conference**

- Yes
- No

**8. Writing a research manuscript**

- Yes
- No

**9. Preparing a research manuscript for submission in a scientific journal**

- Yes
- No
