# Supplementary material for: Confidence, attitude, and practice of scientific research among health professions’ students in the United Arab Emirates
Source: PLoS One. 2024 May 31;19(5):e0304357. doi: 10.1371/journal.pone.0304357 (PMC11142569; doi:10.1371/journal.pone.0304357)
Supplement: S2 File — (DOCX) [file pone.0304357.s003.docx]

| Variable | | median | 25 Percentile | 75 Percentile | P value |
| --- | --- | --- | --- | --- | --- |
| Gender | Female | 24 | 19 | 27 | 0.527 |
|  | Male | 23 | 20 | 27 |  |
| Type of university | Private | 24 | 20 | 27 | 0.241 |
|  | public | 22 | 17 | 26 |  |
| Have you been taught “research methods” during your undergrad study? | No | 22 | 17 | 26 | 0.021 |
|  | Yes | 24 | 20 | 27 |  |
| Do you have prior research experience, or have you been involved in conducting research before? | No | 23 | 18 | 26 | >0.001 |
|  | Yes | 24 | 21 | 28 |  |
| Can you differentiate between different literature resources? | No | 22 | 17 | 26 | 0.001 |
|  | Yes | 24 | 21 | 27 |  |

Table S1. Sociodemographic characters' association with the confidence scores.

Table S2. Sociodemographic characters' association with the practice scores.

| Variable | | median | 25 Percentile | 75 Percentile | P value |
| --- | --- | --- | --- | --- | --- |
| Gender | Female | 3 | 1 | 6 | 0.181 |
|  | Male | 4 | 1 | 7 |  |
| Type of university | Private | 3 | 1 | 6 | 0.037 |
|  | public | 2 | 0 | 5 |  |
| Have you been taught “research methods” during your undergrad study? | No | 1 | 0 | 3 | >0.001 |
|  | Yes | 4 | 2 | 6 |  |
| Do you have prior research experience, or have you been involved in conducting research before? | No | 1 | 0 | 3 | >0.001 |
|  | Yes | 5 | 3 | 7 |  |
| Can you differentiate between different literature resources? | No | 1 | 0 | 4 | >0.001 |
|  | Yes | 4 | 2 | 7 |  |

| Variable | | median | 25 Percentile | 75 Percentile | P value |
| --- | --- | --- | --- | --- | --- |
| Gender | Female | 56 | 49 | 61 | 0.681 |
|  | Male | 56 | 47 | 60 |  |
| Type of university | Private | 55 | 49 | 60 | 0.146 |
|  | public | 57 | 50 | 61 |  |
| Have you been taught “research methods” during your undergrad study? | No | 55 | 49 | 60 | 0.094 |
|  | Yes | 56 | 49 | 61 |  |
| Do you have prior research experience, or have you been involved in conducting research before? | No | 55 | 49 | 60 | 0.136 |
|  | Yes | 57 | 49 | 61 |  |
| Can you differentiate between different literature resources? | No | 55 | 49 | 60 | 0.207 |
|  | Yes | 56 | 49 | 61 |  |

Table S3. Sociodemographic characters' association with the attitude scores.
